# Inoculation with Efficient Nitrogen Fixing and Indoleacetic Acid Producing Bacterial Microsymbiont Enhance Tolerance of the Model Legume* Medicago truncatula* to Iron Deficiency

**DOI:** 10.1155/2018/9134716

**Published:** 2018-10-11

**Authors:** Nadia Kallala, Wissal M'sehli, Karima Jelali, Zribi Kais, Haythem Mhadhbi

**Affiliations:** ^1^Laboratory of Legumes, Center of Biotechnology of Borj-Cedria (CBBC), Hammam-Lif, Tunisia; ^2^Faculty of Science, University of Tunis El Manar, 2092 Tunis, Tunisia

## Abstract

The aim of this study was to assess the effect of symbiotic bacteria inoculation on the response of* Medicago truncatula* genotypes to iron deficiency. The present work was conducted on three* Medicago truncatula* genotypes: A17, TN8.20, and TN1.11. Three treatments were performed: control (C), direct Fe deficiency (DD), and induced Fe deficiency by bicarbonate (ID). Plants were nitrogen-fertilized (T) or inoculated with two bacterial strains:* Sinorhizobium meliloti* TII7 and* Sinorhizobium medicae *SII4. Biometric, physiological, and biochemical parameters were analyzed. Iron deficiency had a significant lowering effect on plant biomass and chlorophyll content in all* Medicago truncatula* genotypes. TN1.11 showed the highest lipid peroxidation and leakage of electrolyte under iron deficiency conditions, which suggest that TN1.11 was more affected than A17 and TN8.20 by Fe starvation. Iron deficiency affected symbiotic performance indices of all* Medicago truncatula* genotypes inoculated with both* Sinorhizobium* strains, mainly nodules number and biomass as well as nitrogen-fixing capacity. Nevertheless, inoculation with* Sinorhizobium* strains mitigates the negative effect of Fe deficiency on plant growth and oxidative stress compared to nitrogen-fertilized plants. The highest auxin producing strain, TII7, preserves relatively high growth and root biomass and length when inoculated to TN8.20 and A17. On the other hand, both TII7 and SII4 strains improve the performance of sensitive genotype TN1.11 through reduction of the negative effect of iron deficiency on chlorophyll and plant Fe content. The bacterial inoculation improved Fe-deficient plant response to oxidative stress via the induction of the activities of antioxidant enzymes.

## 1. Introduction

In plants, iron (Fe) plays an essential role in biochemical processes and plant metabolism such as respiration, photosynthesis, hydroxylation, nitrogen assimilation, symbiotic nitrogen fixation, and regulation of protein stability and as cofactors that carry out electron transfer functions [1, 2]. Despite the abundance of iron in calcareous soil, the bioavailability of Fe can be very low due to high pH and alkaline conditions [3, 4]. Abundance of iron-deficient calcareous soils severely affects plant growth and crop yield adversely [5, 6]. Calcareous soils cover major cultivated land of south Mediterranean lands, which decreased crop growth and yield under low Fe availability. A lot of research showed that the most obvious effect of Fe deficiency is a decrease in the amount of chlorophyll pigments [7, 8]. Therefore, there is a close relationship between plant growth and photosynthesis [7, 9]. In fact, Mann [8] and Ren [9] showed that plants responded strongly to iron deficiency in physiological traits where chlorophyll content and plant biomass were reduced [10]. Hence, in calcareous soils, cultivating Fe-efficient plants is important for maintaining yields while enhancing environmental sustainability.

The Fabaceae family is an important source of proteins [11] providing on average 33% of humans' dietary nitrogen that can reach up to 60% in developing countries [12]. Legumes establish symbiotic interactions with rhizobia leading to the formation of nitrogen-fixing nodules [13]. Nutrient deficiencies such as Fe and P are considered as a critical constraint for the nitrogen fixing and nodule [14–16]. Several studies have shown that the legume–rhizobia symbiosis is particularly sensitive to Fe deficiency [17–19]. In fact, Fe starvation limits root nodule bacterial survival and multiplication, as well as host-plant growth, nodule initiation, and development [20] since it is required for some key proteins involved in nitrogen fixation like nitrogenase, nitrogenase reductase, and leghemoglobin [21].

Indeed, Fe was implicated in cellular reactions of detoxification as a heme moiety, in several antioxidant enzymes like catalases (CAT) and peroxidases (POX), which both ensure the reduction of H_2_O_2_, and as metal cofactor in Fe-superoxide dismutase (Fe-SOD) which converts the superoxide anion to H_2_O_2_. Several research works showed that the induction and protective role of antioxidant enzymes activities under abiotic stress were widely ameliorated in legume-rhizobia by the increase of activities of antioxidant enzymes in nodules which protected biological nitrogen fixation [22–26].

*Medicago truncatula* represents an ideal model legume for the investigation of principal tolerance mechanisms to different environmental constraints such as drought, salt stress [14, 15, 17], and pathogenic agents [27, 28] and for understanding plant–bacteria interactions. Numerous studies of molecular, genetic, proteomic, and physiological aspects have been focused on its symbiosis with* Sinorhizobium* [29, 30]. Therefore, to cope with abiotic stress, many strategies have been developed to improve crop productivity such as microbiological approach involving use of beneficial plant growth promoting rhizobacteria (PGPR) [31]. In fact, rhizobial inoculation has been found as an economical strategy that could produce yield of legumes equal to or better than nitrogen fertilization under drought stress [32] and many studies have reported that the presence and the performance of diverse rhizobial strains were found under stress [24, 33, 34]. It has been recognized that rhizobia could modulate the growth and development of legume crops under stress via antioxidant secretion, if used as an inoculant [26, 35].

In recent years, interest is increasing in the application of plant growth promoting rhizobacteria to ameliorate plant tolerance to abiotic stress and improve plant production. The identification of Fe deficiency tolerant rhizobia has the potential to promote calcareous soils agriculture. Our hypothesis states that plant inoculation could increase plant growth and vigor under Fe deficiency conditions using bacterial strains PGPR performances such as siderophore and auxin production. To date, according to our knowledge, the implication of rhizobia inoculation on* Medicago truncatula* tolerance to iron deficiency was not explored. For that, we considered this research to study the effect of iron deficiency stress on the behavior of different* M. truncatula–Sinorhizobium* associations and to investigate the possibility of improving the tolerance of* Medicago truncatula* to iron deficiency stress by specific adapted microsymbiont inoculation.

## 2. Material and Methods

### 2.1. Bacterial Strains and Growth Conditions

Two strains belonging to the* Sinorhizobium* genus* Sinorhizobium meliloti* (TII7) and* Sinorhizobium medicae *(SII4) [36, 37] from laboratory collection were analyzed to compare their behavior and interaction with* Medicago truncatula* plants under iron deficiency conditions. Bacteria were grown in liquid yeast extract mannitol medium (YEM) [38] in an incubator with controlled growth conditions (150 rpm, 28°C) to obtain a final concentration of 10^9^ cfu ml^−1^.

### 2.2. Production of Indoleacetic Acid (IAA)

To assess the IAA production by bacterial strains, a colorimetric method described by [39] and modified by [40] was used. The bacterial cultures were cultivated in minimal liquid medium for 48 h at 30°C [41]. Aliquots of 250 *μ*l of the bacterial inoculum were used to inoculate 4 ml minimal liquid medium supplemented with tryptophan (0 or 250 *μ*g ml^−1^) and incubated at 30°C until the stationary phase was reached (48–72 h). The bacterial cells were obtained by centrifugation at 8500×g for 5 min, and then 1 ml of the supernatant was added to 200 *μ*L orthophosphoric acid and 4 ml Salkowski reagent [42]. Following incubation at ambient temperature for 20–30 min, the optical density was measured at 535 nm. To calculate the concentration of IAA in each sample, a standard curve ranging from 0.01 to 100 *μ*g ml^−1^ of pure IAA was used for comparison. According to the amount of IAA produced, four distinct levels of IAA production, low production (<15 *μ*g ml^−1^), medium production (between 15 and 30 *μ*g ml^−1^), high production (between 30 and 45 *μ*g ml^−1^), and very high production (>45 *μ*g ml^−1^), were considered.

### 2.3. Siderophore Production

The production of siderophores has been demonstrated by the chromium azurol S test in agar medium. The agar media were prepared according to Schwyn and Neilands [43]: 1/10 of a CAS indicator solution and 9/10 of yeast morphology agar medium. Inoculation was performed by central sting of the agar plates Petri dish. After incubation at 37°C, a red-orange halo appears around the fungal colony attesting to the secretion of siderophores. Measuring the halo's diameter can establish a ration for each strain reflecting the amount of siderophores secreted. The yield of siderophores production (%Ys) was determined as [(halo diameter-colony diameter)/colony diameter]*∗* 100.

### 2.4. Plant Materials and Growing Condition

TN8.20, A17, and TN11.11 seeds were scarified and surface-disinfected (6 min) with sulfuric acid (H_2_SO_4_). After imbibition with distilled H_2_O, seeds were kept at 4°C overnight in darkness. Then, seeds were germinated in Petri dishes for two days at 25°C as described by [29]. After germination, seeds were transferred in autoclaved Agir perlite moistened with distilled water for six days and then to a half-strength aerated sterile nutrient solution in growth boxes for seven days. Similar sized seedlings were selected and cultured as groups of eight plants in nutrient solution (5 L) as described by [44] and modified by [29], containing macronutrients MgSO_4_ (1 mM), KNO_3_ (24 mM), K_2_SO_4_ (0.7 mM), and CaCl_2_ (1.65 mM) and micronutrients as a mixture of salts: MnSO_4_ (6.6 *μ*M), CuSO_4_ (1.56 *μ*M), ZnSO_4_ (1.55 *μ*M), (Na)_2_MoO_4_ (0.12 *μ*M), CoSO_4_ (0.12 *μ*M) 6, and H_3_BO_3_ (4 *μ*M). Plants were inoculated with two rhizobial strains, TII7 and SII4, and the control plants were nitrogen-fertilized with KNO_3_ as a source of nitrogen. Two inoculations were performed: the first was in Agir perlite and the second after the transfer in the growth boxes.

Three treatments were established as follows: control (C: 50 *μ*M Fe(III)-EDTA), direct Fe deficiency (DD: 5 *μ*M Fe(III)-EDTA), and induced Fe deficiency (ID: 50 *μ*M Fe + 10 mM Bic). The solution was renewed every seven days. Plants were placed in a growth chamber for 21 days under controlled conditions (16/8 h light/darkness, temperature was 18°C in the dark with a relative humidity (RH) of 60% and 24°C in the light with a RH of 80% and photosynthetic photon flux density of 300 *μ*mol m^−2^ s^−1^).

### 2.5. Plant Biomass

After 21 days in nutrient solution, plants were separated into shoots, roots, and nodules. Plant material was rinsed with distilled water. The dry weight of each part was determined after drying at 65°C until constant weight for three days and then was used for nutrient analysis. The remaining samples were fixed at -80°C until enzyme activity assays.

### 2.6. Acetylene Reduction Assay

Nitrogenase activity (E.C. 1.7.9.92) was monitored “in situ” by acetylene reduction assay (ARA) using gas chromatography with Porapak T column [45]. ARA measure was performed at the flowering stage corresponding to the optimal nodule activity. Nodule-bearing roots were incubated in 10% C_2_H_2_ atmosphere. After 60 min of incubation, the ethylene formation rate was measured using gaseous phase chromatography. Three replicates of 0.5 ml gas samples were withdrawn from the root atmosphere of each plant, and ethylene production was determined. Pure acetylene and ethylene were used as internal standards [29].

### 2.7. Extraction and Determination of Plant Iron Content

Dry matter of plant parts was weighed and then crushed after drying at 60°C for 72 h. Samples of 25 mg were placed in digestion tube and extracted with 20 ml of nitric acid and perchloric acid (2.5:1, v/v) and brought to 60°C on a hot plate until total desiccation. The solution was then distilled with 20 ml of HNO_3_ (N/7). Finally, the mixture was filtered with Whatman paper. The obtained filtrates are used to determine the extractable iron by means of an atomic absorption spectrophotometer [46].

### 2.8. Chlorophyll Content

Three plants per treatment (with three replicates for each plant) were used to determine the total chlorophyll content of young leaves according to the method of Lichtenthaler [47] with some modifications. A hundred milligrams of small discs from young leaves was incubated in 5 ml 80% acetone in darkness at 4°C for three days (until complete chlorophyll extraction). Then, the total chlorophyll content was determined by a lecture of absorbance at 649 and 665 nm.

### 2.9. The Leakage of Electrolyte

Fragments of 100 mg of the middle part of the freshly cut leaves (three plants/treatment) were placed in assay tubes filled with 10 ml of ultrapure water (deionized). All the tubes were incubated in a water bath for two hours at 32°C. Then, the first electrical conductivity (EC1) of the solution was measured using a type of conductivity Metrohm 712. The same tubes were autoclaved at 121°C for 20 minutes. After cooling to 25°C, the second electrical conductivity (EC2) was measured. The leakage of electrolyte was measured following the formula PE = EC1 / EC2 *∗* 100 [48].

### 2.10. Lipid Peroxidation Assay (MDA)

Lipid peroxidation was determined as described by Cakmak and Horst [49]. 0.2 g of fresh material (three plants/treatment) was homogenized in 4 ml of a 1% (w/v) solution of trichloroacetic acid (TCA). Then, the mixture was centrifuged at 12,000 g for 15 minutes. After that, we added 3 ml of 0.5% (w/v) thiobarbituric acid (TBA) in 20% (w/v) (TCA) to the collected supernatant and the tubes were incubated at 95°C for two hours. The reaction was stopped by placing the reaction tubes in an ice bath. Centrifugation was subsequently done at 9000 g for 10 min. The concentration of the malondialdehyde complex (MDA-) TBA was determined by measuring the optical density of the supernatant at 532 and 600 nm and was expressed using the molar extinction coefficient of 155 mM^−1^ cm.

### 2.11. Antioxidant Enzymes Assays

All operations were performed at 4°C to maintain enzyme activity. Extracts (three plants/treatment) were prepared by homogenizing 200 mg of roots, shoots, and nodules in a mortar with 10% (v/v) polyvinylpolypyrrolidone and 1 ml of phosphate buffer pH 7.8 (50 mM) containing 0.1% (v/v) Triton X-100, 1 mM phenylmethylsulfonyl fluoride as protease inhibitor. Then, the obtained extracts were centrifuged at 13,000 g for 20 minutes and the supernatant was collected for enzymatic activities. Protein content of each sample was measured according to the method of Bradford [50].

Superoxide dismutase (SOD, EC, 1.15.1.1) activity was determined by monitoring the inhibition of photochemical reduction of nitro blue tetrazolium (NBT) at 560 nm [51].

Peroxidase (POX, EC 1.11.1.7) activity was determined by the measurement of the kinetic evolution of tetraguaiacol formation from guaiacol (9 mM) at 470 nm for 1 min with the extinction coefficient (e = 26.6 mM^−1^ cm^−1^) in the presence of 19 mM H_2_O_2_ [52].

Catalase (CAT, EC 1.11.1.6) activity was measured by following the decline in absorbance at 240 nm caused by the catabolization of H_2_O_2_ (10 mM) for 3 min (e = 36 M^−1^ cm^−1^) [53].

### 2.12. Statistical Analysis

Variance analysis of data (three-way ANOVA) was performed using the SPSS 18 program, and means were separated according to the HSD Tukey test at* P* ≤ 0.05. Data shown are means of three replicates for each treatment.

## 3. Results

### 3.1. Strains: Production of Indoleacetic Acid (IAA) and Siderophore

**Figure 1:**
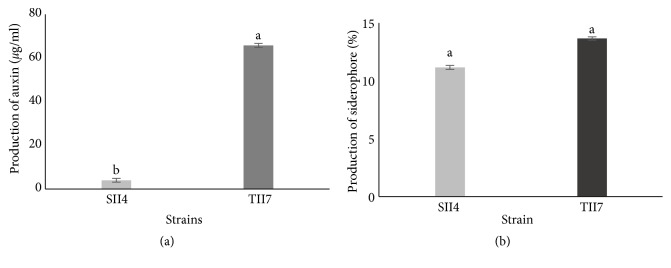
Production of IAA (a) and siderophore (b) of two rhizobial strains: TII7 and SII4.

The results of Figure 1 showed that tested bacterial strains were positive for the production of IAA and showed the ability of synthesis of this phytohormone. Our results showed a clear difference between the two strains in IAA production. In fact, higher IAA concentration was produced by TII7 (65,84 *μ*g/ml) comparing to SII4 strain (4,04 *μ*g/ml). In addition, both TII7 and SII4 were determined as siderophore producers.

### 3.2. Effect of Inoculation on Plant Responses to Iron Deficiency

The analysis of variance for plant growth, root weight and length, iron content, and chlorophyll concentration showed a significant effect for three considered factors strains, genotypes, and treatment and their interaction (Table 1). We noted a high contribution of strains effect to the variance of shoots content of iron and the effect was strongest for chlorophyll content. However, for shoots and roots weight, roots lengths, and roots content of iron, genotype showed the major effect. The treatment effect was manifested remarkably in the variance of shoots content of iron. The interaction effect was lowest for these parameters.

#### 3.2.1. Plant Growth and Root Morphology

**Figure 2:**
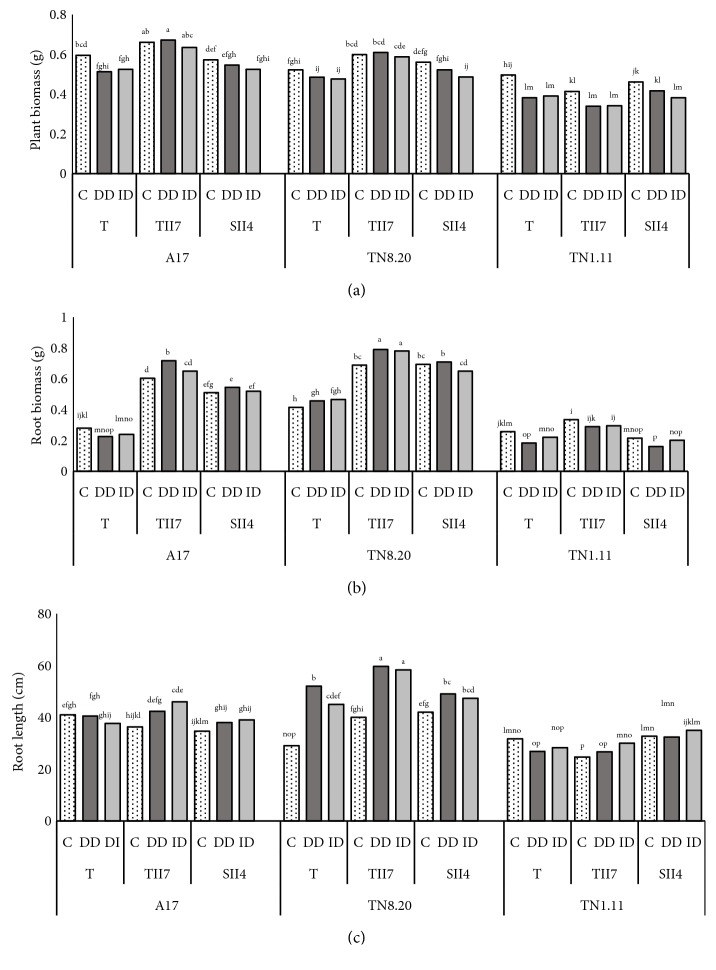
Plant biomass (a), root biomass (b), and root length (c) in* Medicago truncatula* genotypes. T: plants nitrogen-fertilized control, TII7: inoculated with TII7, and SII4: inoculated with SII4, growing in the presence of Fe (C), in the direct iron deficiency (DD), and in the induced iron deficiency (ID) during the treatment period (21 days). Values are the means of eight replicates ± SD at* P*<5%. Graphs denoted with different small letters are significantly different according to the Tukey test.

Results presented in Figure 2(a) showed that iron deficiency affected plant growth of* Medicago truncatula *genotypes. This fact was more detected in TN1.11 genotype under both ID and DD conditions. However, the inoculation of Fe-deficient plants with TII7 and SII4 markedly alleviated the negative effect of iron deficiency on plant growth. As shown in Figure 2(a), this improving effect is clearly identified in A17 and TN8.20 in which we have even observed an improvement in plant deficient growth inoculated with TII7 (-5,2% and -8,3, respectively) compared to the controls (-31% and -26%, respectively). On the other hand, the symbiosis TN1.11-SII4 was found effective in improving the tolerance of this sensitive genotype to iron deficiency. Based on the results of Figure 2(b), we can notice that, under Fe deficiency conditions, the inoculation improved root biomass and length in* Medicago truncatula *genotypes compared to noninoculated plants; this enhancement is more spectacular in plants inoculated with TII7 than those inoculated with SII4. In TN1.11 Fe-deficient plants, the inoculation with SII4 improved plant growth, whereas that with TII7 increased roots length (Figure 2(c)).

#### 3.2.2. Iron Determination

**Figure 3:**
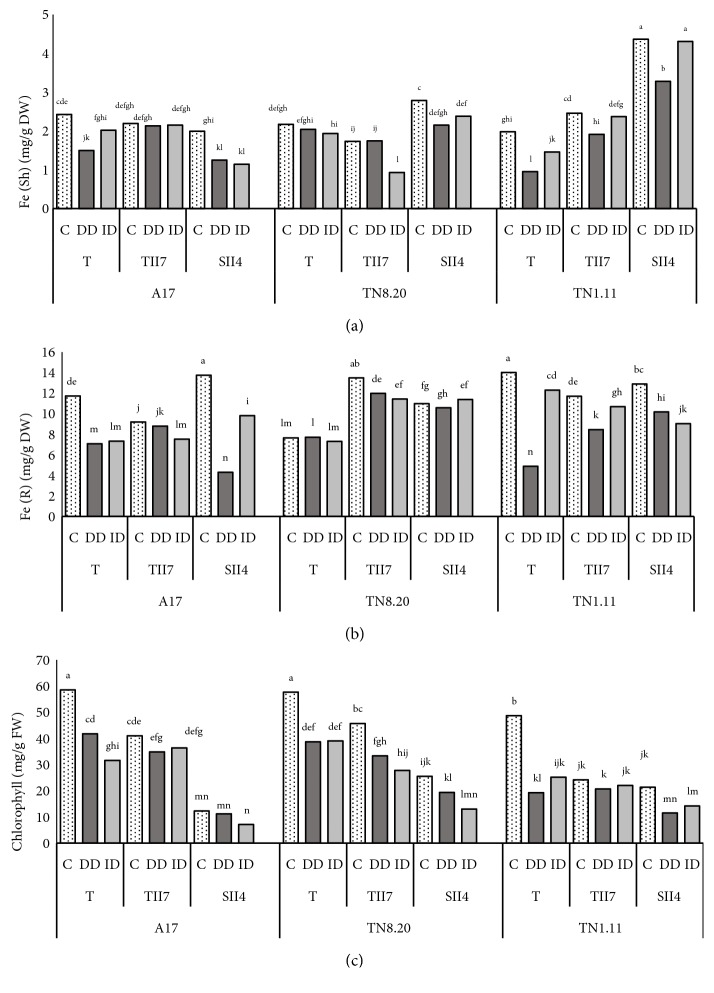
Iron content in shoots (a) and roots (b) and chlorophyll concentration (c) in* Medicago truncatula* genotypes. T: plants nitrogen-fertilized control, TII7: inoculated with TII7, and SII4: inoculated with SII4, growing in the presence of Fe (C), in the direct iron deficiency (DD), and in the induced iron deficiency (ID) during the treatment period (21 days). Values are the means of eight replicates ± SD at* P*<5%. Graphs denoted with different small letters are significantly different according to the Tukey test.

Results presented in Figure 3(a) showed that shoots Fe concentrations were significantly reduced by iron deficiency conditions mainly in A17 and TN1.11 genotypes. Nevertheless, this negative effect is alleviated for the association A17-TII7 cultivated in iron deficiency conditions (DD) by 42% compared to the control. The same behavior was observed for the TN1.11 one inoculated by the two strains TII7 and SII4. Contrary, the inoculation of TN8.20 plants leads to a decrease in shoots Fe content as compared to noninoculated plants. The same responses were observed in roots (Figure 3(b)); we can notice that the inoculation ameliorates roots Fe concentrations under iron deficiency in the association A17-TII7 and in TN1.11-SII4.

#### 3.2.3. Chlorophyll Determination

According to Figure 3(c), iron deficiency caused a significant reduction in leaves chlorophyll content of* Medicago truncatula* genotypes. Considering the genotypic variability, we noted that TN1.11 is significantly affected by this nutritional stress. Under DD treatment, the decrease in chlorophyll content can reach - 41% in TN1.11 while it does not exceed 35% in TN8.20 and 30% in A17. The inoculation moderated the observed decrease in chlorophyll content in all studied genotypes. For example, the reduction of total leaf chlorophyll content became less than -20% in A17, -30% in TN8.20, and -15% in TN1.11 inoculated with TII7 under DD treatment.

### 3.3. Effect of Iron Deficiency on Nodulation and Nitrogen-Fixing Capacity (ARA)

**Figure 4:**
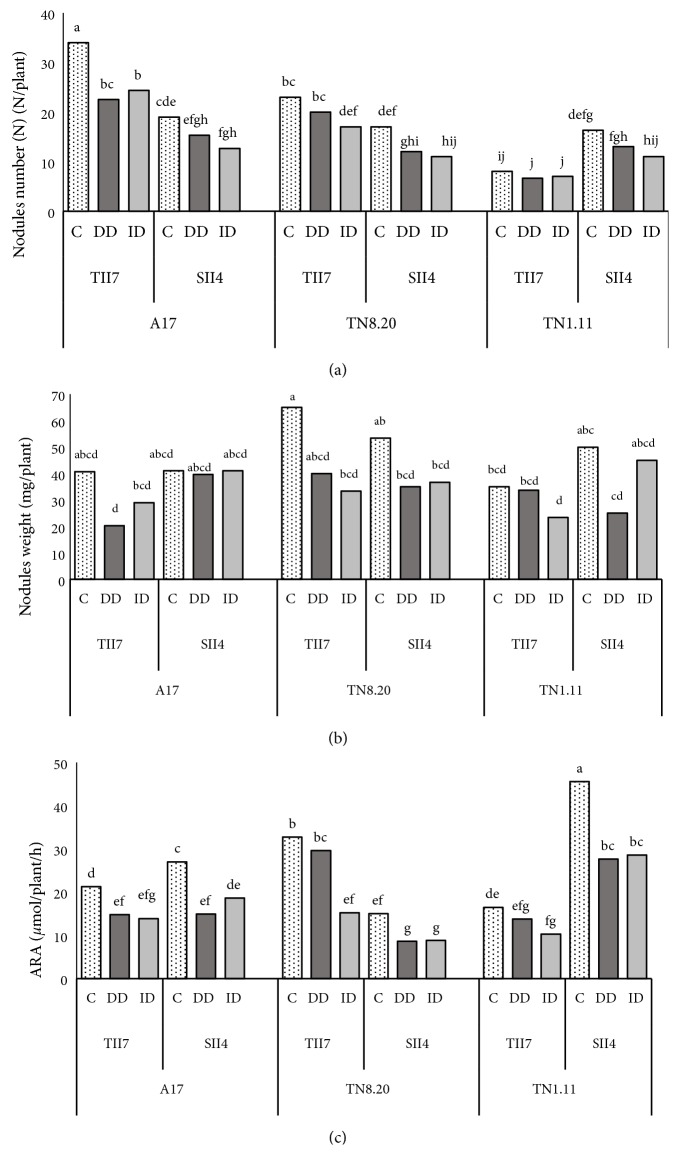
Nodules number (a), nodules weight (b), and acetylene reduction assay (ARA) (c) of* Medicago truncatula* genotypes grown in the presence of Fe (C), in the direct iron deficiency (DD), and in the induced iron deficiency (ID) during the treatment period (21 days). Values are the means of eight replicates ± SD at* P*<5%. Graphs denoted with different small letters are significantly different according to the Tukey test.

As shown in Figure 4, iron deficiency affected the number and the biomass of nodules in* Medicago truncatula* genotypes inoculated with TII7 and SII4. The effect of iron deficiency on nodulation depends on genotype and strain. The analysis of our results lets us deduce that the number and the weight of nodules were higher in the associations A17-TII7 and TN8.20-TII7 which exceed 25% and 22%, respectively, compared to A17 and TN8.20 genotypes inoculated with SII4 and in TN1.11 inoculated with SII4 (42% compared to TN1.11 inoculated with TII7). Moreover, this dramatic effect of iron starvation in number and weight of nodules was concomitant with a decline in nitrogen-fixing capacity (ARA) in Fe-deficient plants. This reduction of nitrogen fixing is more important in TN8.20 inoculated with TII7 (ID) and TN1.11 inoculated with SII4 (DD).

### 3.4. Leakage of Electrolyte and Membrane Damage

**Figure 5:**
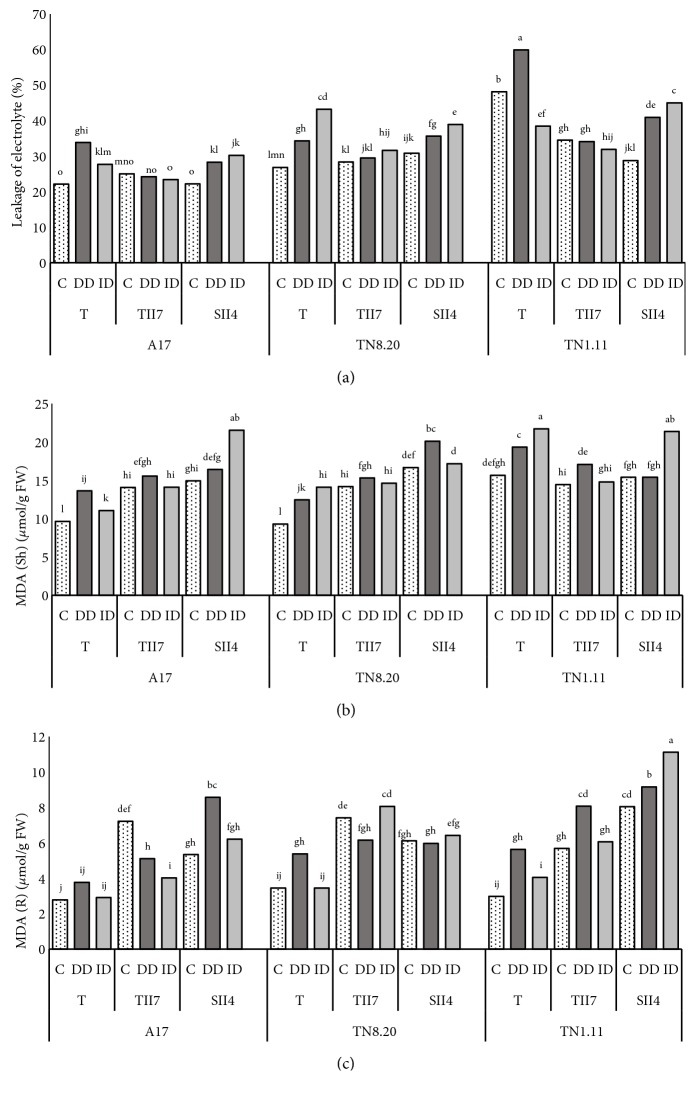
Electrolyte leakage (a), MDA shoots (b), and MDA roots (c) of* Medicago truncatula* genotypes. T: plants nitrogen-fertilized control, TII7: inoculated with TII7, and SII4: inoculated with SII4, growing in the presence of Fe (C), in the direct iron deficiency (DD), and in the induced iron deficiency (ID) during the treatment period (21 days). Values are the means of eight replicates ± SD at* P*<5%. Graphs denoted with different small letters are significantly different according to the Tukey test.

Under iron deficiency conditions, a significant increase in leakage of electrolytes was noted in noninoculated plants of A17, TN8.20, and TN1.11 genotypes (Figure 5(a)). However, the observed increase was mitigated by plants inoculation. It is interesting to note that the best results were observed in plants inoculated with TII7. Indeed, the results showed a significant decrease of leakage of electrolytes for the association A17-TII7 (-3,2%) compared to A17-SII4 (27%) and noninoculated plants (53%) cultivated in iron deficiency conditions (DD). In the same way, Figures 5(b) and 5(c) showed similar findings concerning the beneficial effect of inoculation on shoots and roots MDA concentrations.

### 3.5. Antioxidant Enzyme Responses to Iron Deficiency

The analysis of variance for the antioxidant enzyme activities (SOD, CAT, and POX) showed a significant effect for the considered factors: strains, genotypes, and treatment and their interaction (Table 1). The importance of the effect varied according to the parameter analyzed. We noted a high contribution of strains and genotypes in the variance of these activities mainly in the SOD case.

SOD activity was increased in Fe-deficient plants for all studied genotypes (Table 2). The most pronounced induction was significantly observed in the shoots of the associations A17-TII7 and TN8.20-TII7 compared to A17-SII4 and TN8.20-SII4 (Table 3). However, TN1.11 plants cultivated under DD treatment and inoculated with SII4 showed the highest values of SOD activity. In roots, the best results were found in Fe-deficient TN8.20 plants inoculated with TII7 (+ 76%) compared to those inoculated with SII4 (+ 30%). In nodules, direct iron deficiency stimulated SOD activity in the associations A17-TII7 and TN8.20-TII7. Nevertheless, in the case of TN1.11 plants, the highest increase was found in plants inoculated with SII4.

As compared to control, shoots CAT activity was significantly stimulated by inoculation. The detected increase is more pronounced in A17, TN8.20, and TN1.11 inoculated by TII7 than by SII4 (204%, 353%, and 552%, respectively) under direct iron deficiency. In roots, CAT activity was stimulated in A17 and TN8.20 inoculated with the two strains and we can notice no significant change in TN1.11 genotype. In nodules, CAT activity was stimulated by iron deficiency in deficient plants inoculated with TII7.

Iron deficiency conditions stimulated POX activity in A17 and TN8.20 shoots inoculated with SII4, whereas, in the case of TN1.11, this simulation is detected in Fe-deficient plants inoculated with TII7. In roots, TN8.20 showed a similar behavior compared to leaves. However, in A17, the increase of POX was more spectacular in plants inoculated with TII7 strain (six times greater compared to control under ID treatment). POX activity in nodules increased significantly in response to iron deficiency in TN8.20 inoculated with TII7 (six times greater compared to control).

## 4. Discussion

In the present work, the response of three genotypes of* Medicago truncatula* to iron deficiency was investigated under inoculation conditions. The inoculation was done with two strains: TII7 and SII4. Cultivated in Fe deficiency medium, plants inoculated with TII7 produced the highest plant biomass and root biomass and length. Our results show that rhizobial inoculation of Fe-deficient plants enhanced their biomass production. Documented with several other species [54–56], the plants inoculated with rhizobia showed an increase of growth parameters compared to noninoculated plants. Moreover, it was showed that rhizobial inoculation increased the tolerance of plants under several stressful conditions [24, 57]. This improving effect was due to rhizobial partner. Our results ensure that TII7 and SII4 were able to excrete siderophore and to produce indolic compound (IAA) (Figure 1). The AIA differential production levels between strains can explain the observed improvement of Fe-deficient plant growth, root morphology, and Fe content. Many studies demonstrated that rhizobacteria produced siderophores that can affect the availability and mobility of Fe [58]. In fact, rhizobacteria are characterized by their abilities to take up siderophores de novo synthesized and released [59]. The effectiveness of this ability provides a competitive advantage and confers the efficiency of the resulting symbiosis [18]. Also, IAA is generally known as a growth phytohormone that is a principal regulator of root expansion [60], regulation of root cell elongation and development [61, 62], and functionality in terms of mineral nutrition [63, 64].

A reduction of chlorophyll content was apparent in leaves of* Medicago truncatula* genotypes submitted to DD treatment. The inoculation of Fe-deficient plants mitigated this negative effect only in A17 and TN1.11 and the best results were obtained with the TII7 strain. The same behavior was observed for Fe concentrations; the symbiosis A17/TN1.11-TII7 proved effective in overcoming the negative effect of iron deficiency of Fe content. Velázquez-Becerra and del Carmen Orozco-Mosqueda [65, 66] showed that volatile organic compounds produced by rhizobacteria may serve as signal molecules that induce plant growth by the stimulation of its iron-uptake mechanisms.

In* Medicago truncatula* genotypes, iron deficiency affected nodules number and biomass as well as the nitrogenase activity (detected by the reduction of acetylene reduction activity (ARA)). Our results are confirmed with several other studies, which showed that stressful conditions affect nodule performance. In fact, Slatni [57, 67] and Quin [68] showed that iron deficiency affected soybean nodule performance. Likewise, Mhadhbi [69] found that salt and drought stress inhibited the nitrogen-fixing activity in* Medicago truncatula* genotypes. The decrease in nodule performance observed in Fe-deficient plants can be explained by the fact that iron is an essential mineral for nodule development as it is essential for the activity of nitrogenase and leghemoglobin [70] and through nodule formation, the availability and distribution of iron within the nodule change the role of the symbiotic organ. Rodríguez-Haas [71] controlled the iron distribution in* Medicago truncatula* nodules and their results improved the process of uptake of iron from the rhizosphere and enhanced the hypothesis concerning iron movement within the nodule.

Iron deficiency generates the production of reactive oxygen species (ROS) which in turn induced oxidative stress [72]. Our results showed an increase of leakage of electrolyte and MDA in plants cultivated in Fe-deficient medium. The inoculation by TII7 and SII4 mitigated this increase. According to our results, we can suggest that the decrease in MDA and electrolyte leakage observed in inoculated plants was mainly related to the stimulation of antioxidant enzyme activities (SOD, CAT, and POX). Several works showed that, under stress conditions, the rapid elimination of excessive ROS is essential for the proper functioning of cells and survival of organisms [23]. Our results showed that the activities of antioxidant enzymes in nodules were influenced by iron deficiency and this influence depends on the strain and the genotype.

In conclusion, we demonstrated that iron deficiency affected plant growth parameters of all analyzed* Medicago truncatula *genotypes. Nevertheless, a variability of response was revealed; TN1.11 was more affected than A17 and TN8.20. The inoculation of plants with the two* Sinorhizobium* strains TII7 (*S. meliloti*) and SII4 (*S. medicae*) ameliorates the tolerance of Fe-deficient plants to this nutritional stress. We even observed an improvement of the tolerance of the sensitive genotype TN1.11 to iron deficiency. This enhancement could be related to the capacity of inoculated strains to fix iron via the siderophore production and mainly to produce the auxin phytohormone that was highest within the TII7 strain that showed the best enhancing effect. Both strains stimulate the antioxidant enzyme activities in Fe-deficient plants which could protect deficient plants from the deleterious effect of reactive oxygen species generated under such constraints.

## Figures and Tables

**Table 1:** Results of three-way analysis of the effect of strain (S), genotypes (G), and treatment (T) and their interaction (S*∗*T*∗*G) on shoots weight (ShW: g plant^−1^), roots weight (RW: g plant^−1^), roots length (RL, cm plant^−1^), content of iron in shoots (Fe Sh: mg g^−1^ plant) and roots (Fe R: mg g^−1^ plant), chlorophyll (Chl: mg g^−1^ FW), and antioxidant enzyme activities in shoots (SOD Sh (USOD 10^3^  *μ*g^−1^ protein), CAT Sh (mM H_2_O_2_ min^−1^ mg^−1^ protein), and POX Sh (mM H_2_O_2_ min^−1^ mg^−1^ protein)) and roots (SOD R: USOD 10^3^  *μ*g^−1^ protein, CAT R (mM H_2_O_2_ min^−1^ mg^−1^ protein), and POX R (mM H_2_O_2_ min^−1^ mg^−1^ protein)) in *Medicago truncatula* genotypes.

	Sh W	RW	LR	Fe Sh	Fe R	Chl	SOD Sh	SOD R	CAT Sh	CAT R	POX Sh	POX R
S	48,4^*∗∗*^	1588^*∗∗∗*^	33,2^*∗∗∗*^	247,4^*∗∗∗*^	698,8^*∗∗∗*^	929^*∗∗∗*^	173^*∗∗∗*^	133,6^*∗∗∗*^	34,7^*∗∗∗*^	104,4^*∗∗∗*^	7,47^*∗*^	19,81^*∗∗∗*^
G	785^*∗∗*^	2946,3^*∗∗*^	715,9^*∗∗*^	171,9^*∗∗*^	783,3^*∗∗*^	174^*∗∗*^	6,25^ns^	51,52^*∗∗*^	89,68^*∗∗*^	167,^*∗∗*^	93,58^*∗∗*^	88,78^*∗∗*^
T	135,2^*∗∗*^	2,68^ns^	122,6^*∗∗*^	194,4^*∗∗*^	215,2^*∗∗*^	315^*∗∗*^	63,9^*∗∗*^	5,58^*∗∗*^	81,37^*∗∗*^	5,29^*∗*^	0,52^ns^	49,26^*∗∗*^
S*∗*G	108^*∗∗*^	260^*∗∗*^	48^*∗∗*^	390^*∗∗*^	515^*∗∗*^	52^*∗∗*^	15^*∗∗*^	38^*∗∗*^	72^*∗∗*^	31^*∗∗*^	32^*∗∗*^	100^*∗∗*^
S*∗*T	10^*∗∗*^	17^*∗∗*^	20^*∗∗*^	21^*∗∗*^	225^*∗∗*^	52,6^*∗∗∗*^	66^*∗∗*^	12,8^*∗∗*^	40^*∗∗*^	29,6^*∗∗*^	9,36^*∗∗*^	8,6^ns^
G*∗*T	14,25^*∗∗∗*^	26,7^*∗∗∗*^	78^*∗∗∗*^	26,3^*∗∗∗*^	527,4^*∗∗∗*^	11^*∗∗∗*^	9,25^*∗∗∗*^	7,24^*∗∗*^	30,69^*∗∗∗*^	19,12^*∗∗∗*^	4,59^*∗∗*^	18,8^*∗∗∗*^
S*∗*G*∗*T	3,7^ns^	7,54^*∗∗*^	13,4^*∗∗*^	12,8^*∗∗*^	314,2^*∗∗*^	12,8^*∗∗∗*^	3,69^*∗∗*^	6,31^*∗∗*^	7,74^*∗∗*^	44,22^*∗∗*^	14,65^*∗∗*^	88,5^*∗∗*^

Numbers represent F-value: ^*∗∗∗*^*P*<0.0001, ^*∗∗*^*P*<0.001, and ^*∗*^*P*<0.01.

ns: nonsignificant.

**Table 2:** Superoxide dismutase (SOD: USOD 10^3^  *μ*g^−1^ protein), catalase (CAT: mM H_2_O_2_ min^−1^ mg^−1^ protein), and guaiacol peroxidase (POX: mM H_2_O_2_ min^−1^ mg^−1^ protein) activities in shoots (Sh) and roots of nitrogen-fertilized plants grown in the presence of Fe (C), in the direct iron deficiency (DD), and in the induced iron deficiency (ID) during the treatment period (21 days). Values are the means of eight replicates ± SD at *P*<5%. Values denoted with different small letters are significantly different according to the Tukey test.

Genotypes	Treatment	SOD		CAT		POX	
		Sh	R	Sh	R	Sh	R
A17	C	17,4^c^	61,6^ab^	2,15^e^	243^a^	2,13^ef^	10^c^
	DD	11,8^c^	93^a^	2,17^e^	161^b^	1,48^ef^	22,5^a^
	ID	17,9^c^	91^a^	7,23^e^	108^d^	1^ef^	10,4^c^

TN8.20	C	19,15^c^	44,1^bc^	9,19^e^	22,8^e^	0,6^f^	3,76^de^
	DD	16,71^c^	84^ab^	10,5^e^	30,5^e^	0,46^f^	5,68^d^
	ID	17,09^c^	89^ab^	11^e^	109^cd^	0,32^f^	15^b^

TN1.11	C	17,8^c^	83^ab^	2,64^e^	125,6^bcd^	0,38^f^	13,02^bc^
	DD	16,4^c^	91^a^	2,86^e^	153^bc^	0,37^f^	19,5^a^
	ID	20,2^c^	68^ab^	3,66^e^	24,5^e^	0,63^ef^	13^bc^

**Table 3:** Superoxide dismutase (SOD: USOD 10^3^  *μ*g^−1^ protein), catalase (CAT: mM H_2_O_2_ min^−1^ mg^−1^ protein), and guaiacol peroxidase activities (POX: mM H_2_O_2_ min^−1^ mg^−1^ protein) in shoots (Sh), roots (R), and nodules (N) of *Medicago truncatula *genotypes(G1:A17, G2:TN8.20, and G3: TN1.11) inoculated with two strains (S1: TII7 and S2: SII4) growing in the presence of Fe (C), in the direct iron deficiency (DD), and in the induced iron deficiency (ID) during the treatment period (21 days). Values are the means of eight replicates ± SD at *P*<5%. Graphs denoted with different small letters are significantly different according to the Tukey test.

		SOD			CAT			POX		
		Sh	R	N	Sh	R	N	Sh	R	N
G1S1	C	18,3^bcd^	84^efgh^	38,91^cd^	4,9^efgh^	21,3^fgh^	36,7^def^	0,77^cde^	2,69^f^	2,8^cde^
	DD	37,62^a^	94^defgh^	100^a^	4,9^efgh^	36,4^efghi^	182^a^	0,73^cde^	13^bc^	21,8^a^
	ID	22,8^b^	148^cdef^	110^a^	5,16^efgh^	114,4^b^	101,7^c^	0,97^bc^	7,9^cdef^	0,33^e^
G1S2	C	15,3^bcd^	95^defgh^	37,3^cd^	2,48^fgh^	68,9^cd^	19,6^ghi^	0,71^cde^	3,7^ef^	2,55^cde^
	DD	20^bc^	75^fgh^	35,7^bc^	7,5^def^	62,2^cdef^	24,4^fg^	0,87^cd^	9,2^cde^	2^cde^
	ID	20,5^bc^	125^cdefg^	43,8	9,2^cde^	236^a^	27,8^efg^	1,59^a^	27,6^a^	2,6^cde^

G2S1	C	12,7^cde^	190^c^	18,4^fg^	2,8^fgh^	23,23^efgh^	10,5^hij^	0,79^cde^	10,8^bcd^	1,2^de^
	DD	16,5^bcd^	288^ab^	107^a^	12,8^bc^	12,8^gh^	36,3^def^	1,35^ab^	16,2^b^	8,8^b^
	ID	5,37^e^	328^a^	81,2^b^	5,84^defg^	21,4^fgh^	41^de^	0,79^cde^	28,1^a^	2,7^cde^
G2S2	C	22,3^b^	173^cde^	43,1^bc^	9,9^cd^	20,6^gh^	28,4^efg^	0,43^e^	29,7^a^	4,96^c^
	DD	42,8^a^	211^bc^	36^bc^	19,3^a^	78,6^bc^	28,7^efg^	0,6^cde^	33,9^a^	2,9^cde^
	ID	21^b^	45^gh^	7,4^g^	4,68^fgh^	13,6^gh^	5,3^j^	0,73^cde^	9,4^cde^	2^cde^

G3S1	C	12,9^cde^	177^cd^	44,2^bc^	7,65^def^	9,18^h^	47,8^d^	0,53^de^	7,4^cdef^	4,07^cd^
	DD	11,9^de^	178^cd^	49,7^c^	18,7^a^	13,2^gh^	17,42^ghij^	0,45^e^	7,1^cdef^	2,5^cde^
	ID	12^de^	205^bc^	36,7^bc^	15,2^ab^	15,3^gh^	166^b^	0,54^de^	5,4^def^	1,27^de^
G3S2	C	22,21^b^	25^h^	30,9^def^	1,43^h^	53^cdefg^	5,18^j^	0,6^cde^	6,3^def^	2,7^cde^
	DD	37,3^a^	34^h^	45,8^bc^	4,3^fgh^	64,19^cd^	23,8^fgh^	0,82^cde^	5,7^def^	2,04^cde^
	ID	12,2^de^	40^gh^	21^efg^	2,3^gh^	63^cd^	6,5^ij^	0,43^e^	10,8^bcd^	3^cde^

## Data Availability

The data used to support the findings of this study are available from the corresponding author upon request.
